# Water and soil pollution as determinant of water and food quality/contamination and its impact on female fertility

**DOI:** 10.1186/s12958-018-0448-5

**Published:** 2019-01-13

**Authors:** Justin Rashtian, Diana E. Chavkin, Zaher Merhi

**Affiliations:** 1 Drexel University College of Medicine, Philadelphia, PA USA; 2 HRC Fertility, Los Angeles, CA USA; 3 Department of Biochemistry, Albert Einstein College of Medicine, Bronx, NY 10463 USA; 4 Department of Obstetrics and Gynecology, New York University School of Medicine, 180 Varick Street, Sixth Floor, New York, NY 10014 USA

## Abstract

A mounting body of the literature suggests that environmental chemicals found in food and water could affect female reproduction. Many worldwide daily-used products have been shown to contain chemicals that could incur adverse reproductive outcomes in the perinatal/neonatal periods, childhood, adolescence, and even adulthood. The potential impact of Bisphenol A (BPA), Phthalates and Perfluoroalkyl substances (PFAS) on female reproduction, in particular on puberty, PCOS pathogenesis, infertility, ovarian function, endometriosis, and recurrent pregnancy loss, in both humans and animals, will be discussed in this report in order to provide greater clinician and public awareness about the potential consequences of these chemicals. The effects of these substances could interfere with hormone biosynthesis/action and could potentially be transmitted to further generations. Thus proper education about these chemicals can help individuals decide to limit exposure, ultimately alleviating the risk on future generations.

## Introduction

The constantly increasing pollution of the environment has been one of the greatest concerns for science and the general public in the last few decades. Water and soil pollutants represent two major categories of environmental pollution [1]. Water- and soil-polluting substances are often due to man-made wastes such as household garbage, manufacturing and agricultural wastes, fertilizers used by farmers, oil spills, and radioactive materials [1–3]. Body of water pollution can include rivers, lakes and oceans and it could endanger marine plants and animals. Polluted water and soil represent a serious threat to humans since they can cause acute toxicity, mutagenesis, carcinogenesis, and teratogenesis for humans and other organisms [4–6]. Water pollution can contribute to soil pollution and vice versa [7, 8].

Interestingly and despite governmental legislations, environmental chemicals cross the borders of several countries through business trades of materials, food, and water, exposing humans and animals to them through ingestion, inhalation, and even skin [9]. Some environmental chemicals can disrupt adipogenesis and energy balance thus inducing obesity; additionally, they can alter insulin effect, thus increasing the susceptibility for type 2 diabetes mellitus and cardiovascular system problems [10, 11]. These environmental chemicals now became a major public health concern given that exposure to them, particularly during the sensitive windows of human reproduction, could cause adverse reproductive outcomes (both structural and functional), especially that some have endocrine-disrupting properties [12]. These chemicals can alter multiple physiologic processes and, in case of endocrine disruptors, can interfere with many facets of hormone activity, and their actions depend on the time of exposure as well as the dose and duration of exposure [10].

According to the Endocrine Society, studies have shown that some endocrine disruptors impair germ cell nest breakdown and follicle formation in animal ovarian development, inhibit follicle growth post-natally in animal models, and disrupt steroid hormone levels in humans and animals [10]. These chemicals are also associated with abnormal puberty, irregular cyclicity, reduced fertility, polycystic ovarian syndrome (PCOS), and endometriosis [10]. They can partly mimic or alter the metabolism of the naturally occurring hormones like estradiol (E2), androgen and even thyroid hormones [13].

In this review, we will focus on a subset of well-investigated chemicals that have proven to cause endocrine and reproductive adverse outcomes in females. Thus, this review will present some of the potential impacts, in both humans and animals, of Bisphenol A (BPA), Phthalates, and Perfluoroalkyl substances (PFAS) on female reproduction, in particular on puberty, PCOS pathogenesis, infertility, ovarian function, endometriosis, and recurrent pregnancy loss.

## Search strategy and data extraction

A review was performed for all available basic science, experimental animal studies, and clinical peer-reviewed articles (prospective, retrospective and review articles) published in English from 1995 to date in PubMed. Data were extracted from the text, tables and graphs in the manuscripts. The keywords search used included “Bisphenol A,” “Bisphenol A pathogenesis,” “Bisphenol A and reproduction,” “Bisphenol A and female reproduction,” “Bisphenol A and in-vitro fertilization,” “Phthalates,” “Phthalates pathogenesis,” “Phthalates and reproduction,” “Phthalates and female reproduction,” “Phthalates and in-vitro fertilization,” “Perfluoroalkyl substances,” “Perfluoroalkyl pathogenesis,” “Perfluoroalkyl and reproduction,” “Perfluoroalkyl and female reproduction,” “Perfluoroalkyl and in-vitro fertilization,” “PCOS,” “Infertility,” “Puberty,” “recurrent pregnancy loss,” and “miscarriage” Data on male reproduction were excluded.

### Bisphenol a (BPA)

**Fig. 1:**
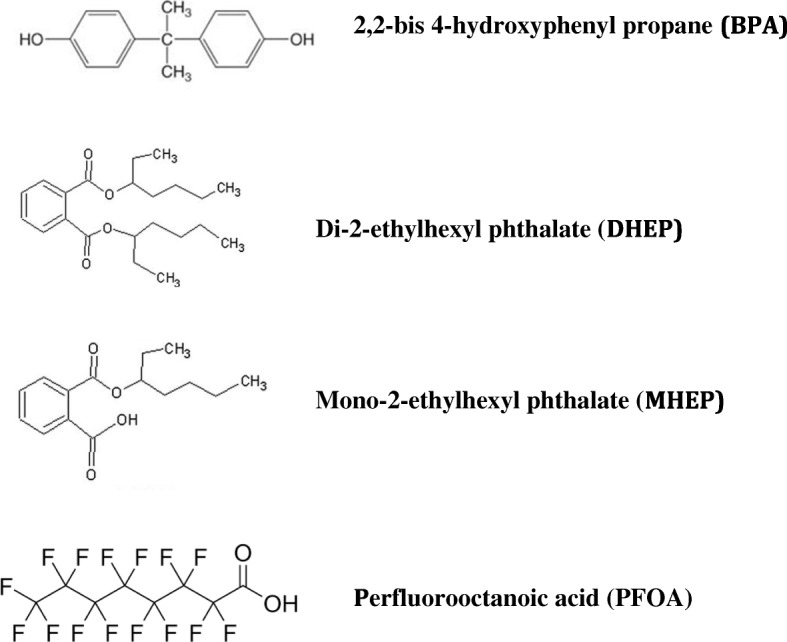
Chemical structure for some of the toxicants discussed in this article

Several epidemiological studies demonstrated significant associations between BPA (chemical structure in Fig. 1) exposure and adverse health outcomes that included reproductive and developmental adverse effects [14]. Many products worldwide used for the production of toilet papers, plastic bottles and containers, envelopes, printer ink, and processed foods such as polycarbonate plastics, epoxy resins, and synthetic polymers can introduce BPA, thus exposing humans (Table 1). In the United States, almost a million tons of BPA are used yearly as a raw material for manufacturing polycarbonate plastic and epoxy resins [14]. Ultimately, BPA reintroduces itself into the aquatic systems via wastewater treatment plants through direct discharge into sewers, sludge, landfills, ground water, river water, canal water, lagoon water, stream water, and estuaries [14]. The monomeric compound BPA is a known endocrine disruptor, which can have significant effects on humans at environmentally low doses. Laboratory studies have shown evidence of its estrogenic effects via affinity binding to E2 receptor (ER), thus mimicking the endogenous effects of E2. Interestingly, BPA could have an antiestrogenic effect via directly binding to androgen receptor [15].

**Table 1:** Examples of products that contain BPA, phthalates and PFAS

Examples of products containing BPA [14, 15]	
Toilet papers	Envelopes
Plastic bottles	Printer ink
Food cans	Processed foods
Toys	Cell phones
Dental fillings	CDs, DVDs
Medical devices	Paint
Examples of products containing phthalates [37]	
Adhesives	Soap
Detergents	Shampoo
Lubricating oils	Lotions
Pharmaceuticals	Nail polish
Solvents	Plastics
Flooring	Medical devices
Examples of products containing PFAS [63, 64]	
Lubricants	Pizza boxes
Paints	Shampoo
Cosmetics	Dental floss
Firefighting foams	Nail polish
Food wrappers and containers	Nonstick cookware
Microwave popcorn bags	Carpets
	Textiles
	Paper
	Contaminated food, water and air
	Red meat, shellfish, eggs and packed snack food

#### Effect of BPA on the reproductive system in female animals

It is well known that the intrauterine milieu is crucial for the future neonatal and adulthood health. In 1999, Howdeshell et al. [16] exposed female mouse fetuses in utero to BPA after feeding pregnant mice BPA dissolved in oil at a dose equivalent to that observed in the environment (2.4 μg/kg). The study found that, in addition to significant changes in the postnatal growth rate of the offsprings, mice had early puberty as reflected by early onset of vaginal opening and the first estrus [16]. Similarly, Honma et al. [17] injected pregnant mice with daily BPA (2 and 20 μg/kg). The female mice exposed to the higher doses of BPA had significantly earlier ages of vaginal opening (a measure of puberty onset) than the controls (injected with oil vehicle only). The first vaginal estrus was also earlier in the BPA-exposed females [17]. These data suggest that exposure to BPA in the pre- and post-natal years could be related to the occurrence of early puberty in the offsprings.

BPA exposure in utero has been shown to alter the morphology of the mammary gland in the offsprings. After exposing mice to environmentally relevant doses of BPA, a study by Muñoz-de-Toro et al. [18] showed that the mammary glands in the BPA-exposed mice were more sensitive to E2 in ovariectomized mice. The terminal end buds were more numerous and had greater areas relative to the areas of the ducts while the apoptotic activity decreased in these mice’s littermates. That study also demonstrated that BPA exposure slowed down the ductal invasion of the stroma, increased clustered ductal epithelial cells for future branching, and increased lateral branching. That study clearly showed that BPA exposure induces abnormal mammary glands development in mice [18]. Markey et al. [19] exposed mice in utero to environmentally relevant doses of BPA (25 and 250 μg/kg body weight) and assessed the histology of the mammary glands. Mice exposed to BPA showed significantly different rates of ductal migration into the stroma, an increase in the percentage of ducts, terminal ducts, terminal end buds, and alveolar buds, as well as an increase in secretory products within the alveoli. Those results showed changes similar to those seen in breast cancer due to BPA and could be explained by the estradiol-mimicking action of BPA [19]. Once again, BPA exposure suggests that it is related to premature or advanced breast development. Whether these findings indicate that BPA could cause early puberty or could increase the risk of breast cancer needs to be investigated in future studies.

Perinatal exposure to BPA has been shown to induce reproductive abnormalities in the female reproductive system. A study [20] showed developmental changes in the reproductive organs of BPA exposed mice where mice were exposed in utero to BPA via pumps implanted into pregnant dams. When they reached adulthood, the female offsprings exposed in utero to BPA had decreased vaginal weight, decreased volume of the endometrial lamina propria, increased expression of endometrial ER-α and progesterone receptor [20]. The findings suggest that these changes could be directly related to BPA’s ability to alter the expression of genes related to estradiol receptor activation.

Data suggest that BPA may interrupt ovarian steroidogenesis by altering steroidogenic enzymes. A study by Zhou et al. [21] investigated the effects of BPA on steroid hormone production in rat ovarian theca-interstitial cells and granulosa cells. In theca-interstitial cells, BPA increased testosterone production and upregulated mRNA expression of the steroidogenic enzymes: 17-α hydroxylase, cholesterol side chain cleavage enzyme, and steroidogenic acute regulatory protein (*StAR*). In granulosa cells, treatment with BPA at particular concentrations caused an increase in progesterone levels and upregulated mRNA expression of cholesterol side chain cleavage enzyme. Interestingly, BPA, in a concentration-dependent effect, inhibited E2 levels and aromatase mRNA expression. These results clearly indicate that exposure to BPA is related to abnormal ovarian function, specifically steroidogenesis. More studies are needed in order to assess whether BPA could be related to abnormal folliculogenesis and anovulation.

Although BPA has endocrine disrupting properties, it also has potential consequences on the gamete genetic quality in both mice and rhesus monkeys [22, 23]. When female mice were given daily oral doses of BPA, exposure during the final stages of oocyte growth elicited detectable meiotic effects. It disrupted, in a dose-dependent manner, chromosome behavior in the oocyte and caused meiotic aberrations [22]. Similar to findings in rodents, another study indicated that maternal levels of BPA analogous to those reported in humans induced detectable effects in the fetal primate ovary [23]. Specifically, early stages of oocyte development in the rhesus monkey were vulnerable to disturbances by BPA, suggesting that fetal exposures may adversely affect the reproductive potential of adult female primates [23]. Despite these studies, it is essential for researchers to obtain a clear understanding of the levels of human BPA exposure as well as the duration of its exposure that could potentially lead to these genetic alterations in human oocytes.

#### Effect of BPA on the reproductive system in female humans

##### Infertility

BPA has been shown to affect female fertility in humans. Several cohort studies have examined BPA levels at different reproductive endpoints in women undergoing fertility treatments. In one study, Ehrlich et al. [24] prospectively measured urinary BPA concentrations in 174 women, aged between 18 and 45, who underwent a total of 237 in vitro fertilization (IVF) cycles. After adjusting for age, body mass index (BMI), day 3 follicle-stimulating hormone (FSH) and smoking, the authors reported that elevated BPA levels were associated with lower number of oocytes retrieved, fewer mature metaphase II oocytes, fewer normally fertilized oocytes, lower serum E2 levels, and a trend for having lower blastocyst formation [24]. These results suggest that BPA is associated with poorer reproductive outcome in infertile women undergoing IVF. Similarly in another study by Mok-Lin et al. [25], the authors measured urinary BPA levels in 84 women who underwent a total of 112 IVF cycles and showed that higher total urinary BPA was significantly correlated with poorer ovarian response, as reflected by fewer oocytes retrieved per cycle and lower serum E2 levels. Higher urinary BPA also correlated with reduced oocyte maturation and lower fertilization rates [25]. In another study by Bloom et al. [26], the authors measured fasting serum BPA levels in 44 women who underwent IVF. Although that study showed that higher BPA levels were significantly associated with lower serum E2 levels per mature follicle, it did not find a significant correlation between BPA and the number oocytes retrieved per IVF cycle [26].

On the other hand, a large and well-designed study by Minguéz-Alarcón et al. [27] found no association between urinary BPA concentrations and IVF outcomes. That prospective cohort study (from 2004 to 2012) was performed at the Massachusetts Fertility Center and included 256 women who underwent 375 IVF cycles. Each woman provided two urine samples prior to oocyte retrieval. General linear mixed models with random intercepts were used to evaluate the association between BPA and IVF outcomes, based on data abstracted from electronic medical records on intermediate and clinical end-points of IVF treatments. The results specifically showed no association between BPA concentrations and peak E2 levels, proportion of high quality embryos, fertilization rates, implantation, clinical pregnancy, or live birth rates per initiated cycle or per embryo transfer. The only significant finding was that there was a relationship between BPA and endometrial wall thickness that was modified by age. Although this was a well-designed study on a large group of women, the authors do agree with the general consensus that data on the relation between BPA exposure and reproductive outcomes remain scares, and that additional research is necessary to clarify BPA’s role in human reproduction.

##### PCOS

The adverse impact that BPA exposure has on fertility in women could be attributed to changes in sex hormone concentrations. Many studies have shown a relationship between BPA and PCOS, one of the most common endocrine disorders in reproductive-aged women associated with hyperandrogenemia. Kandaraki et al. [28] performed a cross sectional study on women with (*n* = 71) or without (*n* = 100) PCOS that were matched by age and BMI. The authors reported that blood BPA levels in the PCOS group were significantly higher than BPA levels in the control group. Even when women were categorized into lean and overweight subgroups, PCOS women in both lean and overweight groups had significantly higher serum BPA levels than the control group. There was also a significant association between serum BPA levels and both testosterone and androstenedione, as well as positive correlation with insulin resistance in the PCOS group. The results of that study suggest that environmental exposure to BPA may play a role in the complex pathophysiology of PCOS [28].

Another study by Takeuchi et al. [29] found similar results. The authors measured serum BPA levels in 26 women with normal menstrual cycles (control group) (17 of whom were obese); 19 women with PCOS (6 of whom were obese); 7 women with hyperprolacinemia, and 21 women with hypothalamic amenorrhea. Both obese and normal weight women with PCOS had significantly higher BPA levels than normal weight controls. The study also reported that serum BPA levels were positively correlated with serum testosterone (total and free), androstenedione, and dihydroepiendrosterone (DHEA) concentrations in all study participants. Therefore, this study further shows a relationship between BPA and androgen levels further implying that BPA may play a role in the pathophysiology of PCOS [29]. Interestingly, in vitro exposure to BPA at low doses does not affect granulosa cell steroidogenesis, while at supra-physiological concentrations, BPA alters progesterone and estradiol synthesis and significantly reduces the mRNA and protein expression levels of 3β-HSD, CYP11A1 and CYP19A1 [30]. Additionally, in one study, urinary BPA concentrations did not correlate with CYP19 mRNA expression levels in granulosa cells of women who underwent IVF [31].

Although studies have shown consistency in the relationship between BPA and PCOS, the association is still vague since PCOS is a complex endocrine problem associated with elevated androgen and insulin resistance. Whether BPA has a causal relationship rather than only correlation with PCOS or one of its features, such as elevated androgens or hyperinsulinemia, remains to be determined.

Not only BPA affects steroid production (as mentioned above in the animals section), but the reverse could be true, i.e., steroids, such as androgens, can affect BPA levels. Since high levels of BPA are observed in hyperandrogenemic women with PCOS, a study [32] investigated the effect of testosterone on BPA metabolism. Ovariectomized female rats were injected subcutaneously with increasing doses of testosterone propionate daily for 2 weeks after which serum BPA levels were measured. The results showed that serum BPA increased with testosterone propionate administration in dose-dependent manner. The authors also quantified the enzyme reaction of BPA glucuronidation in the rat liver. Their results showed that the ratio of glucuronide in the rats was significantly reduced in a testosterone dose-dependent manner. Additionally, the relative mRNA expression of UDP-glucuronosyltransferase 2B1 (*UGT2B1*) showed a testosterone dose-dependent decrease. The ratio of BPA glucuronidation and *UGT2B1* mRNA levels were significantly lower in environment with elevated testosterone. That study concluded that the clearance of BPA might be slowed down in the presence of high levels of testosterone, thus potentially explaining the elevated serum BPA levels in hyperandrogenic women with PCOS.

##### Puberty

The relationship between BPA levels/exposure and puberty in humans has been evaluated in several studies and the results have been controversial. In a cross sectional study, Wolff et al. [33] did not find an association between urinary BPA levels and premature puberty in 9-year-old girls (*n* = 192). In another prospective cohort study of girls aged between 6 and 8, Wolff et al. [34] did not find an association between urinary BPA levels and breast or pubic hair development. On the other hand, Qiao et al. [35] showed that serum BPA levels were significantly elevated in girls with precocious puberty compared to controls and higher serum BPA levels were positively correlated with increased uterine and ovarian volume. A recent review article [36] reported that out of 19 studies, only 7 showed a correlation between BPA and puberty. Taken altogether [36], although data in animal models showed a relationship between BPA exposure and early puberty (above section on animals), available data to date in humans do not show a clear role for BPA in pubertal development in humans due to the conflicting results among all studies examined.

##### Recurrent pregnancy loss

Exposure to BPA may be associated with recurrent pregnancy loss. A study [37] showed that serum BPA levels in women with a history of three or more consecutive first-trimester miscarriages (*n* = 45) were significantly higher compared to serum BPA levels in 32 healthy women (no history of live birth and infertility). In a case-control study in eastern China [38], total urinary BPA concentrations were measured in 102 women with recurrent miscarriages and 162 control women (all participants aged 20–40 years). The creatinine-adjusted BPA levels were significantly higher in women with recurrent miscarriages compared to control women. Additionally, higher level of urinary BPA was significantly associated with a 3–9 times increased risk of recurrent miscarriages [38]. Clearly, more prospective well-designed studies are needed to better assess the relationship between BPA and recurrent pregnancy loss.

### Phthalates

Phthalates are a group of chemicals mainly used to give flexibility and resilience to plastics. Phthalates exist in many products that are used daily such as adhesives, detergents, lubricating oils, medical devices, pharmaceuticals, solvents, flooring, soap, shampoo, lotions, and nail polish (Table 1). These chemicals are then readily released into the environment where they were found in some foods and indoor dust as well as water sources and sediments. Humans get ultimately exposed to phthalates through ingestion, inhalation, and even skin contact although dietary sources have been considered the major route of exposure [39]. In this review, we will focus on di-2-ethylhexyl phthalate (DEHP). Following ingestion, DEHP gets metabolized into mono-2-ethylhexyl phthalate (MEHP), which is considered the active biomarker of DEHP exposure (chemical structures in Fig. 1). Studies have suggested that DEHP is an agonist of the peroxisome proliferator-activated receptor (PPAR) and pregnane x receptor (PXR), and it has been shown to alter the synthesis of estrogens and androgens [40].

#### Effect of phthalates on the on the reproductive system in female animals

There is accumulating evidence from experimental animal studies suggesting that phthalates exert reproductive toxicity by targeting the ovary [41]. Phthalates has been demonstrated to disrupt folliculogenesis, steroidogenesis, oocyte maturation and embryonic development thus leading to reduced fertility [42, 43]. In a study by Wang et al. [44], the treatment of mouse ovarian follicles with MEHP inhibited the growth of antral follicles and showed an increase in reactive oxygen species (ROS) levels. In that study, the authors then measured the activities of various key antioxidant enzymes including copper/zinc superoxide dismutase (SOD1), glutathione peroxidase (GPX), and catalase (CAT) as well as the expression of key cell-cycle regulators. They showed that, compared to control ovarian follicles, MEHP induced oxidative stress by disrupting the activity and the expression of the antioxidant enzymes SOD1 and GPX, but not CAT. MEHP also inhibited the expression of *Ccnd2, Ccne1, Cdk4,* and *Bcl-2*, but increased *Bax* expression. Thus, the authors suggested that phthalate metabolites may lead to decreased expression of cell-cycle regulators and antiapoptotic regulators, while leading to increased expression of proapoptotic factors; all this leading to the inhibition of follicular growth [44]. Li et al. [45] studied the apoptotic effects of different concentrations of MEHP on rat ovarian granulosa cells in vitro. High doses of MEHP inhibited granulosa cell viability and increased apoptosis rates. Transcription factors and pathways involved in inducing apoptosis (increase in CASPASE3 activity and Bax/Bcl2 ratio) also demonstrated significantly higher expression with increased MEHP exposure [45]. These results suggest that MEHP might play a role in inducing apoptosis in ovarian granulosa cells potentially altering ovarian function such as steroidogenesis and folliculogenesis.

Inada et al. [46] isolated secondary follicles from female rats and cultured them with different concentrations of MEHP. The diameters, viability, and apoptosis of these follicles were measured, along with the steroid hormone levels in the culture media. High concentration of MEHP treatment was significantly associated with decreased viability of follicles and increased apoptosis of granulosa cells. Additionally, progesterone levels were markedly increased while androstenedione, testosterone, and E2 levels were significantly decreased. The results also suggested that MEHP could inhibit the conversion of progesterone to androstenedione. That study further indicates that MEHP induces ovarian toxicity in rats both by suppressing follicular development and by causing abnormal changes in steroidogenesis [46]. Guo et al. [47] administered DEHP to pregnant mice and then measured serum progesterone and E2 levels, the number and size of corpora lutea following histology, mRNA expression levels of steroidogenic enzymes, ovarian CD31 protein (marker of endothelial cells) by immunohistochemistry, and plasma prostaglandin F2-alpha levels. Their results revealed that treatment with DEHP significantly inhibited progesterone secretion in a dose-dependent manner, increased plasma prostaglandin F2-alpha levels, downregulated *CYP11A, 3β-HSD,* and *StAR,* reduced the numbers and sizes of corpora lutea, and inhibited CD31 expression of corpora lutea [47]. These findings suggest that, likely via these combined mechanisms, DEHP significantly inhibits luteal function of pregnant mice in vivo.

Hannon et al. [48] cultured neonatal ovaries from CD-1 mice with either DEHP or MEHP, and antral follicles from adult CD-1 mice with MEHP. In the neonatal ovaries, the results demonstrated that MEHP decreased the percentage of germ cells and increased the percentage of primary follicles by decreasing phosphatase and tensin levels and increasing phosphorylated protein kinase B levels. In the antral follicle cultures of adult mice, MEHP decreased testosterone, estrone, and E2 levels by downregulating mRNA levels of the enzymes: 17alpha-hydroxylase-17,20-desmolase, 17beta-hydroxysteroid dehydrogenase, and aromatase. The study showed that MEHP accelerated folliculogenesis via PI3K (a pathway that regulates primordial follicle quiescence and activation) overactivation, and inhibited steroidogenesis [48]. Niermann et al. [49] exposed pregnant CD-1 mice to DEHP and reported significant increase in the preantral follicle count in the pups. The later fertility of the DEHP-treated pups was also affected since it took them longer time to get pregnant compared to controls [49]. These findings indicate that MEHP could cause a serious concern on ovarian function by compromising folliculogenesis and steroidogenesis.

#### Effect of phthalates on the reproductive system in female humans

##### Infertility

As mentioned earlier, evidence from toxicological studies in animals has demonstrated that phthalates could adversely impact fertility through effects on folliculogenesis, steroidogenesis, oocyte maturation and embryonic development, but human data are scarse. Concentrations of eight phthalate metabolites in 110 follicular fluid and urine samples were collected from women (*n* = 112) attending an infertility clinic in China were quantified and the results showed that follicular fluid and urinary metabolite concentrations of MEHP were not associated with any IVF parameters such as peak E2 levels, number of oocytes retrieved, number of mature oocytes, fertilization rates, number of good-quality embryos and rate of blastocyst formation [50]. However, that study was limited by the small sample size, which may not have adequate power to detect significant associations. Interestingly, among women with a history of infertility, the molar sum of urinary DEHP was significantly lower in women who conceived after IVF compared to those who did not [51]. Whether women pursuing fertility treatments take precautions to avoid exposure to environmental toxins, to improve treatment outcomes needs to be explored. Thus, given the prevalence of phthalates exposure, further larger studies are needed to elucidate the potential hazard on female reproduction in the setting of infertility treatment.

In a prospective study, Messerlian et al. [52] evaluated the association between 11 urinary phthalate metabolites and antral follicle growth in a study that included 215 infertile women. The higher levels of urinary phthalate concentrations negatively correlated with antral follicle count indicating that phthalates are associated with lower ovarian reserve in infertile women [52]. Interestingly, among women with a history of infertility, urinary DEHP levels were significantly lower in women who conceived after assisted reproductive technology compared to women who did not get pregnant [51]. Mural granulosa cells from 48 patients undergoing IVF were treated with increasing concentrations of dibutyl-phthalate in vitro following which gene microarray analysis was performed [53]. When compared with untreated cells, exposure to high doses of dibutyl-phthalate resulted in significant differences in expression of 346 annotated genes (151 were upregulated and 195 were downregulated). The main functional annotations affected were associated with cell cycle and mitosis thus indicating that acute treatment with high concentrations of dibutyl-phthalate alters gene expression pathways mainly associated with the cell cycle. Reinsberg et al. [54] collected human luteinized granulosa cells from women undergoing IVF and cultured them with varying concentrations of MEHP in the presence of FSH, hCG and cAMP after which they assessed steroidogenesis. MEHP suppressed aromatase expression and E2 production in a dose-dependent manner. MEHP did not, however, change the production of progesterone [54].

A study [55] assessed whether urinary concentrations of metabolites of phthalates and phthalate alternatives were associated with IVF outcome in 136 women where participants provided one to two urine samples per cycle during controlled ovarian stimulation and then before oocyte retrieval. Urinary concentrations of the sum of DEHP and other phthalate metabolites were negatively associated with the number of total oocytes retrieved, total number of mature oocytes, total number of fertilized oocytes, and good quality embryos but none of the urinary phthalate metabolite concentrations were associated with a reduced implantation, lower clinical pregnancy or lower live birth rates [55].

Literature to date indicates that phthalates could inhibit the size of the growing antral follicle pool and could potentially impair fertility and fecundity. There is a need of further investigations pertaining to the impact of phthalates on the human oocyte and follicular development.

##### PCOS

As of today, there are no studies focusing on the relationship between DEHP and MEHP with PCOS. In one study, 52 subjects with PCOS had lower urine concentrations of phthalate metabolites compared to subjects without PCOS [56]. In another study including 244 girls, the sum of phthalate metabolites was protective against PCOS in adolescence where there was a negative association of phthalate with PCOS and of phthalate with serum anti-Mullerian hormone [57]. Future studies are needed to confirm these preliminary findings and determine if DEHP and MEHP may have a role in the pathogenesis of PCOS.

##### Recurrent pregnancy loss

There is preliminary contentious evidence showing that early pregnancy may be adversely affected by DEHP exposure. The first study to show this association included Danish women (*n* = 128) who reported increased risk of early pregnancy loss with higher urinary concentrations of the DEHP metabolite MEHP [58]. In another study that included women (*n* = 256) undergoing assisted reproduction, there was an increased conception cycle-specifc urinary concentrations of total sum of DEHP and individual DEHP metabolites were associated with biochemical pregnancy loss [59]. On the other hand, menstrual cycle–specific estimates of urinary phthalate metabolites in 221 women were not associated with detrimental alterations in follicular-phase length, time to pregnancy, or early pregnancy loss; rather DEHP metabolites were associated with reduced early loss [60]. Thus there is no clear consensus on whether DEHP/MEHP are related to early pregnancy loss and there is a need for such studies.

##### Endometriosis

There is a possible link between phthalate esters and endometriosis. Cobellis et al. [61] collected blood and peritoneal fluid samples from 55 women with endometriosis and 24 age-matched women without endometriosis. Women with endometriosis had significantly higher plasma DEHP concentrations than control women and the majority of women with endometriosis had detectible levels of DEHP and/or MEHP in the peritoneal fluid. Similarly, another study by Kim et al. [62] showed that the urinary concentration MEHP, mono (2-ethyl-5-oxohexyl) phthalate and mono (2-ethyl-5-carboxyphentyl) phthalate, were significantly higher in women with endometriosis compared to women without endometriosis. Another prospective study by Kim et al. [63] showed that 97 women with advanced-stage endometriosis had significantly higher plasma levels of MEHP and DEHP compared to 169 control women [63]. On the other hand, Itoh et al. [64] found no significant association between endometriosis and 6 urinary different phthalate monoesters in infertile Japanese women who had laparoscopy for diagnosis of endometriosis after adjusting for urinary creatinine. In that study, the control group subjects were categorized as stages 0–1 endometriosis (*n* = 80) and the experimental group subjects were categorized as stages 2–4 endometriosis (*n* = 57).

Interestingly, a study [65] treated human endometrial stromal cells with different concentrations of DEHP and assessed ROS generation, expression levels of antioxidant enzymes, alteration of MAPK/NF-κB signaling and hormonal receptors. DEHP increased ROS generation and decreased expression of superoxide dismutase (*SOD*), glutathione peroxidase (*GPX*), heme oxygenase (*HO*), and catalase (*CAT*). DEHP induced p-ERK/p-p38 and NF-κB mediated transcription and induced estradiol receptor-α expression in a dose-dependent manner. That study suggests that DEHP may be associated with the development of endocrine-related disease such as endometriosis.

Finally, consistent with the substantial body of evidence from animal studies, more human studies are emerging linking phthalates with altered female reproductive system. Considering that phthalate exposure is nearly universal, these results may have important clinical and public health relevance.

### Perfluoroalkyl substances (PFAS)

Perfluoroalklyl substances (PFAS) are a group of substances that are ubiquitous in the environment and are thought to have detrimental with long lasting effects on metabolic, endocrine, and reproductive as well as pubertal and sexual development in humans. PFAS include substances such as perfluorooctanoic acid (PFOA) (chemical structure in Fig. 1), perfluorododecanoic acid (PFDoA), perfluorononanoic acid (PFNA), perfluorodecanoic acid (PFDA) and perfluoroundecanoic acid (PFUnDA), perfluorooctane sulfonate (PFOS), perfluorohexane sulfonic acid (PFHxS). Many of these substances are commercially prevalent substances used in industrial processes and products such as lubricants, paints, cosmetics, firefighting foams and food packaging materials (Table 1). Because they are so prevalent with daily exposure, they are detected globally in humans and animals. Many perfluoroalkyl acids (PFAAs) are found in different human tissues following exposure through ingestion of contaminated food, water, and air. There is evidence that serum PFOA concentrations are increased after the intake of red meat, shellfish, eggs and packed snack food and to a lesser extent with vegetables and poultry intake. Breast milk is also found to be a source of PFAS exposure for infants. There have also been reported findings of PFAS in umbilical cord blood indicating that there may be a prenatal risk [66].

#### Effect of PFAS on the reproductive system in female animals

PFAAs have attracted attention in recent years for their environmental ubiquity and toxicity. White et al. [67] assessed the relationship between PFOA exposure and mammary gland development in mice. They found that when given to pregnant mothers at a 5 mg/kg/d dose, there was stunting of mammary epithelium development independent of changes in body weight. In a later study, White et al. [68] demonstrated that there could be a severe effect on the development of the mammary glands even when exposure occurs only through breast milk. When given to pregnant mice, PFOA caused a lasting effect on the mammary glands in female offsprings. This suggests that PFOA is sequestered into breast milk in mice and that early postnatal exposure to PFOA have the potential to permanently alter mammary gland development. Additionally the authors assessed the effect of mammary gland development in a multigenerational study after exposure to drinking water. Lactation morphology was compromised in second-generation offsprings and following chronic drinking water exposure at 5 ppb (leading to serum PFOA levels of 60–90 ng/mL). In addition to its effect on glandular development, PFOA has been reported to delay pubertal timing in female mice as reflected by delay in vaginal opening [69]. Thus the adverse consequences of developmental exposures to perfluorooctanoic acid (PFOA) seem to be established in mice, and they include impaired development of the mammary glands.

PFDoA exposure may affect the expression of genes related to E2 production and E2 signaling in pubertal female rats. Shi et al. [70] reported that when rats were orally given PFDoA, there was a significant decrease in body weight, decrease in serum E2 levels, increase in cholesterol levels and altered expression of genes responsible for steroidogenesis such as *StAR* protein, cholesterol side chain cleavage enzyme and 17 beta-hydroxysteroid dehydrogenase. There was also a decrease in ER-α and ER-β expression in the ovary as well as a decrease in ER-β RNA levels in the uterus. Serum levels of LH and FSH levels were not affected by PFDoA exposure. There was no effect on sexual organ weight or on age at first estrous cycle or on the ovarian or uterine histology. These data indicated that PFDoA does not necessarily affect puberty in rats but has an effect on steroidogenic enzymes as well as on E2 production and E2 receptors.

#### Effect of PFAS on the reproductive system in female humans

##### Infertility

A number of studies have suggested that PFAS exposure can seriously impair the reproductive health in humans, both in fertility and infertility setting. A study by Fei et al. [71] showed that women with higher serum PFOA levels had higher rates of subfecundity and longer time to achieve a pregnancy. They also reported that women with the highest PFOA exposure had increased rates of menstrual cycle irregularities. In the Maternal-Infant Research on Environmental Chemicals (MIREC) Study, a cohort study of 2001 women recruited before 14 weeks of gestation in 10 cities across Canada, the investigators reported that, after adjustment for potential confounders, PFOA and PFHxS were associated with approximately 10% reduction in fecundability per one standard deviation increase; however, no significant association was observed for PFOS [72]. In addition, the odds of infertility increased by 31% per one standard deviation increase of PFOA and by 27% per one standard deviation increase of PFHxS, while no significant association was observed for PFOS [72].

Studies on the exposure to PFAAs and female fertility have provided conflicting results. Jorgensen et al. [73] evaluated human fecundity by measuring time to pregnancy in women from different geographical populations (Greenland, Poland and Ukraine) representing varying PFAS exposure and pregnancy planning behaviors. They assessed the association between serum levels of PFOA, PFOS, PFHxS and PFNA in those women and rates of infertility (defined as time to pregnancy more than 13 months). They found that higher PFNA levels were associated with infertility in the pooled sample and specifically in women from Greenland. PFNA effect on infertility was weaker for women from Poland and Ukraine. Although they found that PFNA levels could be associated with infertility, they did not find this association for other PFAS such as PFOS, PFOA or PFHxS. In a follow up study, Bach et al. [74] investigated the association between PFAS and infertility in additional populations. In a pooled analysis including parous and nonparous women, they found that the fecundibility rates were lower in women with higher levels of PFOS and PFOA. PFOS was not associated with higher rates of infertility but there was a trend for an association between infertility and PFOA in parous women. Bach et al. [75] found no association between PFAA levels in maternal serum prior to 20 weeks gestation and diagnosis of infertility in nulliparous women (*n* = 1372). This is consistent with their previous findings [74] where only a trend for an association between infertility and PFOA in parous but not nulliparous women was reported. Interestingly, there is evidence showing that follicular fluid levels of perfluorinated compounds in women undergoing IVF have a detrimental effect of oocytes fertilization capacity with subsequent decrease in the number of embryos transferred [76].

Altogether, there is a mild evidence that exposure to PFAS, even at low levels, may reduce fecundability and that environmental exposure to PFAS impairs female fecundity by delaying time taken to conceive.

##### PCOS and reproductive hormones

A number of studies have suggested that the effects of PFAS on reproductive health and development are mediated by their effect on the hormonal milieu. In a case control study, subjects with PCOS (*n* = 52) had significantly higher geometric mean serum concentrations of PFOA and PFOS compared to controls (*n* = 50) [56]. That study suggest that women with PCOS could have a different environmental contaminant profile. Barrett et al. [77] found that certain PFAS are associated with ovarian hormonal changes in certain populations of reproductive-aged women. They measured daily salivary levels of E2 (calculated mean follicular levels) and progesterone (calculated mean luteal levels) as well as daily serum levels of PFAS (including PFOS and perfluoroctanoic acid) in young healthy regularly cycling women (*n* = 178) in a single menstrual cycle. They found that in nulliparous, but not parous, women that PFOS and perfluorooctanesulfonic acid levels were inversely associated with E2 and progesterone levels. Tsai et al. [78] evaluated the association between PFAS serum concentrations and reproductive hormones in young Taiwanese adults and adolescents (between 12 and 30 years old) and found that serum POFA, PFOS and PFDA levels were negatively associated with serum levels of SHBG, FSH and testosterone--associations that were strongest in females aged between 12 and 17. Maissonet et al. [79], in Avon Longitudinal Study of Parents and Children (ALSPAC), found that prenatal exposure to PFASs can affect the hormonal milieu even later in life. They assessed pregnant women (*n* = 72) at 16 weeks gestation for serum levels of PFAA and then measured total testosterone and SHBG in their daughters at 15 years of age. They found that total testosterone concentrations were higher in daughters with prenatal exposure to PFOS or PFOA but not PFNA. SHBG was not affected by exposure to any PFAAs in utero. These results indicate that exposure to certain PFAA (PFOS, PFOA, PFHxS) in utero can lead to alterations in a woman’s testosterone levels later in life.

In brief, PFAS seem to be related to parity and could affect steroidogenesis. These potential alterations could lead to abnormally elevated androgens and might theoretically contribute to the complex pathogenesis of PCOS.

##### Recurrent pregnancy loss

Equivocal findings have been reported for the association between PFAS and miscarriages. A prospective study assessed PFAS and pregnancy loss in couples (*n* = 501) that were followed daily from preconception through the 7th week post-conception. There was no significant association between pregnancy loss and any of the 7 PFASs that were quantified: 2-N-ethyl-perfluorooctane sulfonamide acetate (Et-PFOSA-AcOH); 2-N-methyl-perfluorooctane sulfonamido acetate (Me-PFOSA-AcOH); perfluorodecanoate (PFDeA); PFNA; perfluorooctane sulfonamide (PFOSA); PFOS; and PFOA. Limitations of that study were that women used home pregnancy test kits, and that pregnancy loss was documented by conversion from a positive to a negative pregnancy test, onset of menses or clinical confirmation. Similarly, another study showed no association between serum PFOA or serum PFOS levels with miscarriage rate [80]. In a prospective study of miscarriage in a population exposed to high levels of PFOA and PFOS, there was little evidence of association with serum levels of PFOA and limited evidence of association with serum levels of PFOS [81]. As of today, it is hard to draw from the evidence to date a clear conclusion between the relationship between PFAS and pregnancy loss.

## Conclusion

**Fig. 2:**
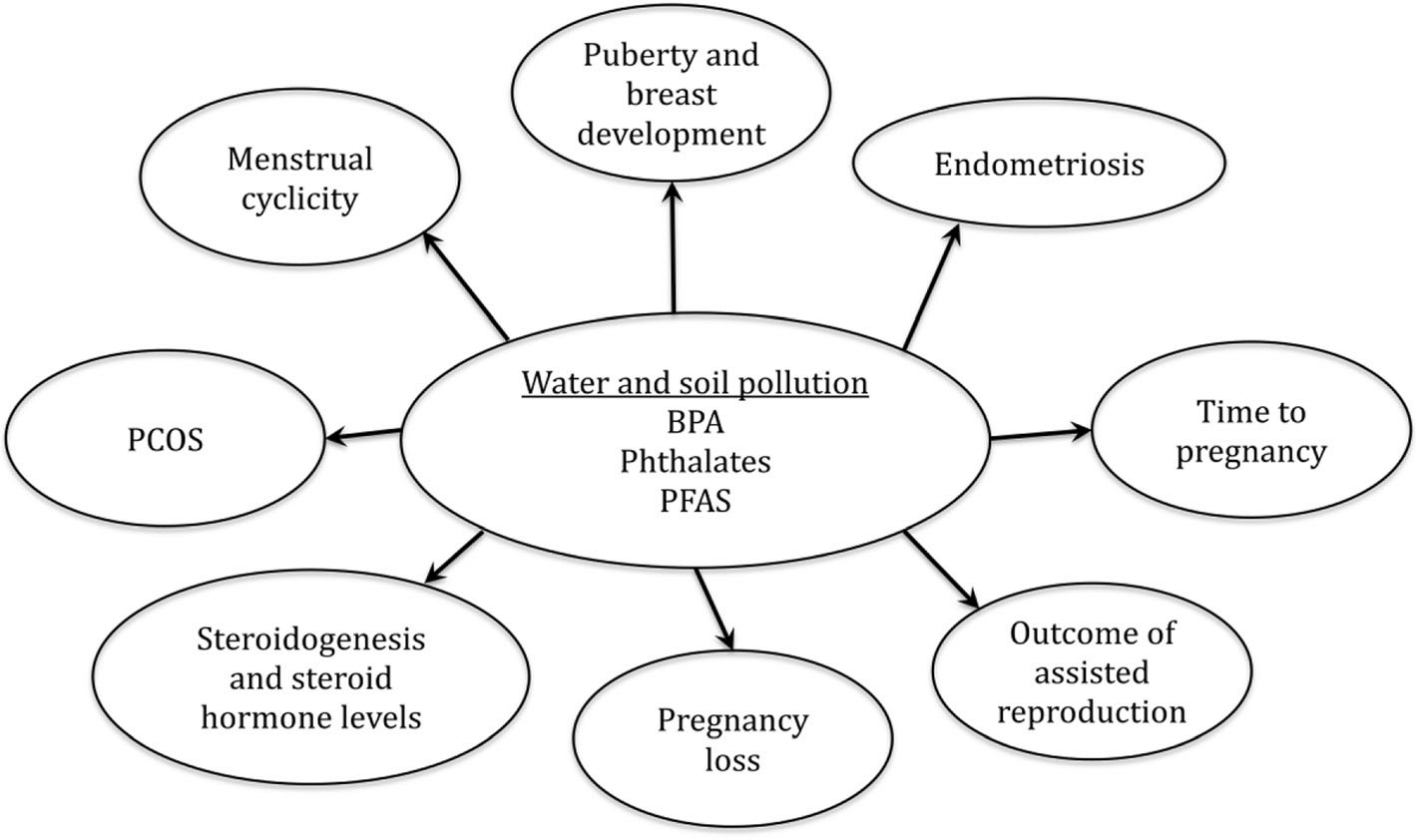
Potential reproductive hazards of BPA, phthalates and PFAS

Environmental contaminants including endocrine disruptors are a worldwide problem and are hidden players in reproductive health. The aim of this review was to provide greater clinician and public awareness about the potential consequences of some of these chemicals (Table 2 and Fig. 2) whose effect could be transmitted to further generations. Effective protection from chemical exposures requires governmental leadership, environmental education, and social action. Thus proper education about these chemicals can help individuals limit their exposure to these chemicals (at least to a certain extent) in food and water, ultimately alleviating the risk on future generations. Finally, there is a greater need for longitudinal studies with emphasis on precaution and prevention as well as multigenerational studies in humans.

**Table 2:** Reproductive effects of BPA, phthalates and PFAS

Reproductive effects of BPA	
- Related to early onset of puberty ▪ Earlier age of vaginal opening in *female mice* [17]. ▪ Increased uterine and ovarian volume in young female humans [33]. - Alter mammary gland development ▪ Larger and more abundant terminal end buds in relation to ducts, decreased apoptotic activity, slowed ductal invasion of the stroma, and increased lateral branching in mammary glands of female mice [18]. ▪ Altered rates of ductal migration into the stroma, increase in the percentage of ducts, terminal ducts, terminal end buds, and alveolar buds; an increase in secretory products within the alveoli of female mice [19]. - Associated with poorer outcome following assisted reproductive technology ▪ Lower number of oocytes retrieved, fewer mature metaphase II oocytes, fewer normally fertilized oocytes, lower serum E2 levels, and a trend for having lower blastocyst formation in women [24]. ▪ Poorer ovarian response, as reflected by fewer oocytes retrieved per cycle and lower serum E2 levels; reduced oocyte maturation and lower fertilization rates in women [25].	- Role in the pathophysiology of PCOS ▪ Associated with higher levels of both testosterone and androstenedione, as well as insulin resistance, in women with PCOS [28]. ▪ Additionally, higher levels of DHEA in women with PCOS in another study [29]. - Alters ovarian steroidogenesis (increase testosterone production) ▪ PCOS studies: androgen levels (seen above) [28, 29]. ▪ IVF studies: E2 levels (seen above) [24, 25] - Associated with increased risk of recurrent miscarriages ▪ Higher BPA levels in women with 3 or more consecutive 1st trimester miscarriages than in healthy woman [35]. ▪ Higher urinary BPA levels correlated with a 3–9 times increased risk of recurrent miscarriages in women [36].
Reproductive effects of phthalates	
-Increase reactive oxygen species ▪ MEHP induced oxidative stress by disrupting the activity and the expression of the antioxidant enzymes SOD1 and GPX; also inhibited the expression of *Ccnd2, Ccne1, Cdk4,* and *Bcl-2*, but increased *Bax* expression in the ovarian follicles of female mice [42]. -Alter cell cycle regulation and apoptosis ▪ High doses of MEHP inhibited granulosa cell viability and increased apoptosis rates in the ovaries of female mice [43, 44]. -Negatively correlated with ovarian reserve ▪ Higher levels of urinary phthalate concentrations negatively correlated with antral follicle count in infertile women [50]. - Elevated in endometriosis ▪ Women with endometriosis had significantly higher levels of phthalate metabolites in three different studies [58–60].	-Alters ovarian steroidogenesis and folliculogenesis ▪ Under high concentrations of MEHP, progesterone levels were markedly increased while androstenedione, testosterone, and E2 levels were significantly decreased in female mice [44]. ▪ DEHP significantly inhibited progesterone secretion in a dose-dependent manner, increased plasma prostaglandin F2-alpha levels, downregulated *CYP11A, 3β-HSD,* and *StAR,* reduced the numbers and sizes of corpora lutea, and inhibited CD31 expression of corpora lutea in female mice [45]. ▪ MEHP decreased testosterone, estrone, and E2 levels by downregulating mRNA levels of the enzymes: 17alpha-hydroxylase-17,20-desmolase, 17beta-hydroxysteroid dehydrogenase, and aromatase in female mice [46].
Reproductive effects of PFAS	
-Alters mammary gland development ▪ Relationship between PFOA exposure and mammary gland development in female mice [64, 65]. -Delays puberty ▪ Delay in vaginal opening female mice [66] -Alters ovarian steroidogenesis ▪ Decrease in ER-α and ER-β expression in the rat ovary [67] ▪ Decrease in serum E2 levels in female rats [67] -Alters reproductive hormone levels ▪ Serum POFA, PFOS and PFDA levels were negatively associated with serum levels of SHBG, FSH and testosterone in female humans [74]. ▪ Total testosterone concentrations were higher in daughters with prenatal exposure to PFOS or PFOA in female humans [75]. -Increased rates of infertility ▪ Women with higher serum PFOA levels [68] and higher PFNA levels [70] had higher rates of subfecundity ▪ Women with higher serum PFOA levels had longer time to achieve a pregnancy [68]	-Menstrual irregularities ▪ Women with the highest PFOA exposure had increased rates of menstrual cycle irregularities [68]. -Association with miscarriage risk ▪ No association between serum PFOA or serum PFOS levels with miscarriage rate in women [76]. ▪ Little evidence for an association between serum levels of PFOA and miscarriage rate in women [77].

## Abbreviations

BMI: Body Mass Index
BPA: Bisphenol A
CAT: Catalase
DEHP: Di-2-Ethylhexyl Phthalate
DHEA: Dehydroepiandrosterone
E2: Estradiol 2 receptor
FSH: Follicle Stimulating Hormone
GPX: Glutathione Peroxidase
IVF: In-Vitro Fertilization
MEHP: Mono-2-Ethylhexyl Phthalate
PCOS: Polycystic Ovary Syndrome
PFAAs: Perfluoroalkyl acids
PFAS: Perfluoroalkyl Substances
PFDA: Perfluorodecanoic Acid
PFDoA: perfluorododecanoic acid
PFHxS: Perfluorohexane Sulfanoic Acid
PFNA: Perfluorononanoic Acid
PFOA: Pefluorooctanoic Acid
PFOS: Perfluorooctane Sulfonate
PFUnDA: Perfluoroundecanoic Acid
PPAR: Peroxisome Proliferator-Activated Receptor
PXR: Pregnane X Receptor
ROS: Reactive Oxygen Species
SOD1: copper/zinc Superoxide Dismutase
